# Hospital Administration

**Published:** 1896-12-26

**Authors:** 


### The Institutional Workshop.

### HOSPITAL ADMINISTRATION.

### THE HOSPITAL REFORM ASSOCIATION.

It is difficult to regard with gravity the initial effort made by the Hospital Reform Association to obtain a little elementary information on the subject which it has tak[illegible] hand. It has issued a series of six questions, apparently to its own members, to which it has received replies from twenty individuals, whose fitness for the task would seem to vary almost as greatly as does the length of their replies, which range from the monosyllabic to the diffuse. We rise from a perusal of the answers vouchsafed by these reformers with the feeling that one among their number—namely, Mr. Timothy Holmes—has some knowledge of the subject, and knows his own mind, although his project may not be very feasible, but that many of the rest are in a hopeless muddle, and that some of them fail to grasp the real inwardness of the subject on which they have undertaken to introduce reforms.

The first question put is, "In your experience do persons well able to pay a doctor's fee avail themselves of the benefit of the out-patient department?" To this question Mr. Timothy Holmes answers: "I have not much experience on the subject. What I have would lead me to think that the abuse in this respect of the O. P. department is not great in London." Mr. Walter Rivington says: "Personally, I did not find this to be the case to any extent in the surgical O. P. department at the London Hospital, or at the London Dispensary, Spitalfields." Professor Victor Horsley, however, says: "Yes, I have turned away many myself," and in this he is supported by witnesses from Croydon, Portsmouth, Dudley, Hatfield, Redhill, Dover, &c. Which is, of course, very convincing. Dr. Saundby, of Birmingham, however, boldly says "No," and generally it may be said that so far as the inquiry has drawn answers from people in any way responsibly connected with the large metropolitan hospitals, the answers have been like unto that of Balaam, who, instead of cursing, blessed them altogether; although the men in the country, as is their wont, seem much impressed with the wickedness of London and all its ways.

We naturally turn with the greatest interest to the answers received to the inquiry "If you believe that abuse of that department exists, in what way would you propose to remedy it?" This is coming to the point, and it really is by the answers given to this question that the Hospital Reform Association must stand or fall, for the people who give these answers are not men collected from the highways and byways, but are the men who actually form the association itself.

Dr. Knowsley Sibley says, "Limit the number admitted and receive only those with letters from doctors and other competent persons." Who are "competent persons"? and how does Dr. Sibley think the work of the medical profession will be lightened or the hospitals be better managed by forcing people to beg for hospital letters from medical men?

Mr. T. R. Atkinson, who says he knows of cases of abuse, can only suggest that, "When the public realise that there is such a thing as hospital abuse they will be prepared to discourage any except poor persons going to the out-patient departments"; but he suggests that, 'for the benefit of an intermediate class who desire the opinion of a hospital doctor in their case but cannot pay the fees of a consultant, a consulting department might be attached to every hospital, to which the general practitioner might bring or send such cases. For such consultations a fee of five shillings ought to be charged." Can it be seriously believed that the addition of a paying consulting branch to a charity out-patient department would in any way lighten the present load or lessen the crush of patients or the hurry-scurry with which they are said to be passed through the consulting-rooms? We venture to say that this proposal shows considerable ignorance of the actual condition of affairs at most of our hospitals. Why should a poor fellow who has been to half the doctors in his neighbourhood be asked for five shillings for a consultation on his case, while a drunken fellow out of the street—poor, indeed, according to the definition, but utterly undeserving—walks in and gets advice and medicine all for nothing?

Even Professor Horsley, the only hospital surgeon in London who speaks positively as to the existence of abuse, can only suggest as follows: "By appointment of inspectors to inquire into the status of persons suspected to be taking advantage of the charity, and publishing in the department the results of such inquiries."

But can we believe that the ordinary hospital out-patient would care a single farthing for having his name posted up among the thousand other names which would be included in such a list, not one of which would ever be looked at? In little country towns people may care about their neighbour's affairs, but the London out-patient wants to see his doctor and get his medicine as quickly as possible, but about nothing else does he care, except, maybe, the warmth of the waiting-room; and to think that he will be deterred from coming by the knowledge that his name will be posted up on some notice-board is mere childishness. Who ever looks at a notice-board in these days?

The inherent difficulties of the question are shown by the evidences of softness of heart which are displayed by even the most truculent opponents of the out-patient system when they come face to face with the idea of turning patients away. Dr. Welsford would cure the evils "by abolishing the department altogether." No doubt that is his desire, but when he puts pen to paper, and has to sign his name, he sees the difficulty, and he hedges, adding: "A receiving-room should be established to replace the O. P. department, where patients could be seen once and either taken in if seriously ill, or referred to private medical practitioners; in the case of applicants who are obviously too poor to obtain medical assistance the officer on duty may have discretionary power to give them an order for a definite time in the receiving-room." This speaks well for Dr. Welsford's kindness of heart; but the working of the scheme would be exactly what happens now at some of the hospitals, an admission officer standing among a crowd of applicants and giving admissions to the most plausible among them, a plan certainly very bad for the class of cases who are often the most deserving, the cases of obscure disease to whom the general practitioners they have consulted have failed to give relief.

We are quite clear that most of those who have answered these questions have failed to get a real grasp of the situation as it exists at the present time, and their suggestions are in many cases feeble in the extreme. Mr. Timothy Holmes, however, stands on a somewhat different footing. He has evidently studied the subject, and has formulated some ideas. Whether they would work, however, is another question, to which we may return later, but in any case, if carried out, they would quite revolutionise the generally accepted notions of an out-patient department. Mr. Holmes would so far approximate the customs of the out-patient department to those of Harley Street as to make each patient come at a fixed time by appointment; which we should imagine would be exactly what the well-to-do patient would most like. But he would "admit no one except on the recommendation of some medical authority." This must mean that the would-be out-patient must go to a private medical man in the first instance, which would not be difficult to the well-to-do, but might be distinctly hard on the poor man. We can quite admit that under such circumstances the out-patient department of many a hospital might be more orderly than at present, and that the patients might receive more careful attention; but whether the proper persons would receive that attention is another matter. Sweep out the poor and the out-patient departments will be all the sweeter, but it may well be questioned whether they will be so completely doing their proper work. and in view of the fact that the very reason for the existence of the Hospital Reform Association is the assumed injury done by the hospitals to the general practitioner, it is worthy of note that Mr. Holmes' sympathy seems all to be with the poor ill-used out-patient and not with the general practitioner at all. In the reply given to this question by Mr. Holmes, not one word is said as to any means to be taken with the object of rejecting those whose pecuniary circumstances render them unfit objects for the receipt of charitable relief, and in answer to the next question he definitely says: "I look on the pecuniary circumstances of the patient as a matter of relatively subordinate importance." This does not seem to make for harmony in the formation of a programme for the protection of the general practitioner.

### ADDENBROOKE'S HOSPITAL.

The deadlock between the University of Cambridge and the authorities of Addenbrooke's Hospital is a vexatious one, and one that is likely seriously to interfere with the prospects of the Cambridge University medical school. The question is whether the hospital shall appoint the University Professor of Surgery on its staff, or the University shall choose its professors from among the medical officers of the hospital. There can be no doubt that the present condition of affairs is extremely anomalous, and it is hard to believe that even as regards medicine it can work well for the school. For surgery it is quite out of the question. By an arrangement between the University and the hospital, Professor Clifford Allbutt is at present enabled to use the cases in the hospital, or such of them as can be moved into the theatre, for purposes of clinical teaching, and it has been hoped on the University side that it might be possible to make some such arrangement in regard to surgery. To teach surgery on another man's cases would, however, be a very lame performance, and we may be allowed seriously to question the propriety of the proceeding from the patients' point of view. A surgeon to a hospital is master over his house surgeon and his dressers, and is responsible for their proceedings; but he could hardly be held responsible for the proceedings of so exalted a personage as a University professor, who might, for example, differ from him in his estimation of the importance of the antiseptic methods employed in the treatment of a case. No! It would never do. Whatever happens, the man who teaches must be the one who treats the cases and is responsible for them. The matter is eminently one for compromise. No doubt the University people think it incompatible with their dignity that the subscribers to the hospital should have the first choice in the election of the professor of surgery, in so far as they would elect the staff from among whom the University professor would be appointed, and, no doubt, on the other side the hospital authorities hesitate to hand over the treatment of their patients to men who would be entirely outside their own control. Moreover, there is the Act of Parliament to be considered. The advantages of a compromise are too obvious to be put on one side. It is quite clear that for clinical teaching the teacher should, if not a member of the staff, at least have similar duties; while there can be no doubt that by the association of the University with the hospital a very high class of men would be drawn to the staff of Addenbrooke's Hospital, much to the advantage of that institution.

